# Toward cost-effective staffing mixes for Veterans Affairs substance use disorder treatment programs

**DOI:** 10.1186/s12913-015-1175-7

**Published:** 2015-11-23

**Authors:** Jinwoo J. Im, Ross D. Shachter, John W. Finney, Jodie A. Trafton

**Affiliations:** Management of Innovation Program, Daegu Gyeongbuk Institute of Science and Technology, Daegu, 711-873 South Korea; Department of Management Science and Engineering, Stanford University, Stanford, CA 94305 USA; Center for Health Care Evaluation, VA Palo Alto Healthcare System, Menlo Park, CA 94025 USA; Department of Psychiatry and Behavioral Sciences and Center for Health Policy, Stanford University School of Medicine, 795 Willow Road (152-MPD), Stanford, CA 94305 USA

**Keywords:** Substance use disorder (SUD), Staffing, Optimization, Cost-benefit analysis

## Abstract

**Background:**

In fiscal year (FY) 2008, 133,658 patients were provided services within substance use disorders treatment programs (SUDTPs) in the U.S. Department of Veterans Affairs (VA) health care system. To improve the effectiveness and cost-effectiveness of SUDTPs, we analyze the impacts of staffing mix on the benefits and costs of specialty SUD services. This study demonstrates how cost-effective staffing mixes for each type of VA SUDTPs can be defined empirically.

**Methods:**

We used a stepwise method to derive prediction functions for benefits and costs based on patients’ treatment outcomes at VA SUDTPs nationally from 2001 to 2003, and used them to formulate optimization problems to determine recommended staffing mixes that maximize net benefits per patient for four types of SUDTPs by using the solver function with the Generalized Reduced Gradient algorithm in Microsoft Excel 2010 while conforming to limits of current practice. We conducted sensitivity analyses by varying the baseline severity of addiction problems between lower (2.5 %) and higher (97.5 %) values derived from bootstrapping.

**Results and conclusions:**

Compared to the actual staffing mixes in FY01-FY03, the recommended staffing mixes would lower treatment costs while improving patients’ outcomes, and improved net benefits are estimated from $1472 to $17,743 per patient.

## Background

The U.S. Department of Veterans Affairs (VA) is tasked with providing comprehensive health care services to Veterans within a capitated global annual budget set by the U.S. Congress. In order to provide more effective and cost-effective services, VA is interested in understanding how organizational structure, staffing, and treatment process impact patients’ health outcomes. VA treats a large number of Veterans for substance use disorder (SUD) in specialized SUD treatment programs (SUDTPs) at an annual cost of more than $350 million [1]. For example, in fiscal year 2008 (FY08), 403,117 Veterans seen in the VA healthcare system were diagnosed with SUDs; 133,658 of them received services within VA’s specialized SUDTPs [2]. VA operates four types of SUDTPs: inpatient, residential, intensive outpatient and standard outpatient. Inpatient programs provide acute, in-hospital care, detoxification and medical stabilization services. Residential and intensive outpatient programs both focus on psychosocial stabilization and helping patients with active SUDs achieve a period of early abstinence, but residential programs provide a more structured setting for patients with specific risk-factors. Residential programs provide less medicalized services and longer lengths of stay than inpatient programs. Intensive outpatient programs treat patients at least 3 days per week and provide at least 3 h of services per patient per day [3]. Standard outpatient programs treat patients 1 to 3 days per week and provide lower intensity ambulatory addiction treatment services, generally focusing on relapse prevention and maintenance of initial treatment gains in stabilized patients.

Staffing accounts for the majority of specialty SUD treatment costs. Thus, understanding how staffing mix within SUDTPs impacts the benefits and costs of specialty SUD services is key to improving the effectiveness and cost-effectiveness of treating this patient population. Some evidence-based treatments (e.g., opioid agonist treatment) [4] may only be delivered by staff with higher levels of clinical training or they may be delivered with greater adherence to protocol by staff with more clinical training. Moreover, graduate-level training has been found to be associated with modest but beneficial effects on psychotherapy outcomes and retention in treatment [5]. For these reasons, more highly trained employees may be more effective in treating SUD patients, but they also tend to be more expensive.

Given the differences in the treatment focus and services required by patients in each of the four types of SUDTPs, their staffing mixes can be quite different (e.g., 6 % psychologists for inpatient vs. 1 % for outpatient). This study aims to demonstrate how cost-effective staffing mixes for each type of VA SUDTPs can be defined empirically for the treatment programs.

## Methods

### Optimization

We identified recommended staffing mixes through the process of optimization. Optimization involves searching through a set of feasible solutions for one that achieves the best value of an objective function. In our case, our objective was to maximize net benefits per patient within each type of SUD treatment program. Each solution corresponds to a staffing mix, the percentage of staffing hours provided by each staff type to a patient in a particular treatment program. A feasible staffing mix is one that satisfies a set of constraints to ensure that it is reasonable and conforms to the ranges observed in practice. Once potential solutions, objective function, and constraints are defined, standard algorithms can be used to determine the best feasible solution. We constructed the optimization problems in Microsoft Excel 2010 and solved them by using the solver function with the Generalized Reduced Gradient (GRG) algorithm to determine recommended staffing mixes for each type of treatment program. The detailed descriptions of the GRG algorithm can be found in the following literatures [6–8].

Optimization problems have been formulated to tackle diverse health care issues. Some examples include scheduling for bladder cancer patients [9, 10], determining resource allocation in HIV prevention programs [11–13], and creating a portfolio of screening and contact tracing for endemic diseases [14]. Optimization problems also have been formulated to identify a better staffing mix for high technology companies [15–17], call centers [18, 19], and healthcare providers [20, 21]. However, we could not find any study that addressed optimal mixes of different types of health professionals for the context of SUDTPs.

We set the percentages of 12 different types of staff (e.g., psychiatrist, addiction therapists, and clerks, Table 1) in a treatment program as the decision variable matrix (*x*). We hypothesized that varying the composition of staffing *x* alters the benefits from the treatment program *B(x)* in addition to changing staffing costs *C(x)*. We only considered staffing costs as a cost factor because other costs, such as facility overhead, can be regarded as fixed and unaffected by the decision variables *x* and staffing costs are the major cost drivers for SUDTPs. We determined the recommended staffing mix x* by solving the following optimization problem:

**Table 1:** Staff types and FY01-FY03 average wages

Staff type	Staff	FY01-FY03 average wage ($)	
		Hourly	Annual
Prescribers	MD	75.74	157,534.22
	RESMD	22.99	47,826.54
	PRACT	43.70	90,891.88
Psychosocial Rehabilitators	PSYCH	46.66	97,047.07
	SOCWK	33.85	70,405.23
	ADTHR	30.15	62,716.61
	OCTHR	33.67	70,039.89
Nurses	RN	37.25	77,487.39
	LVN	22.82	47,472.50
Support Administrators	CLERC	17.84	37,113.40
	AIDES	23.08	48,008.63
Trainees	PDTR	10.87	22,609.48

MD: % psychiatrists or mds; RESMD: % resident physicians or fellows; PRACT: % nurse practitioners or physicians assistants; PSYCH: % psychologists; SOCWK: % social workers; ADTHR: % addiction therapists; OCTHR: % recreational/occupational therapists; RN: % registered nurses; LVN: % licensed vocational/practical nurses; CLERC: % clerical staff; AIDES: % aides/technicians; PDTR: % paid trainees

1 $$ \max\ W \cdot B(x)-C(x) $$

2 $$ s.t.\ {\displaystyle \sum\ x=1} $$

3 $$ \min \left({x}_{observed}\right)\ \le\ x\ \le \max \left({x}_{observed}\right) $$

4 $$ \min \left({\left({x}_{trainee}/{x}_{supervisor}\right)}_{observed}\right)\ \le\ \left({x}_{trainee}/{x}_{supervisor}\right)\le \max \left({\left({x}_{trainee}/{x}_{supervisor}\right)}_{observed}\right) $$

5 $$ \min \left({L}_{observed}\right)\ \le\ L(x)\le \max \left({L}_{observed}\right) $$

6 $$ \min \left({H}_{observed}\right)\ \le\ H(x)\le \max \left({H}_{observed}\right) $$

7 $$ \min \left({B}_{reasonable}\right)\ \le\ B(x)\le \max \left({B}_{reasonable}\right) $$

Equation 1 indicates that this optimization problem aims to find a staffing mix to maximize net benefits per patient from a treatment program. The term *W* denotes conversion factors to transform benefits derived from treating a patient into monetary values. Some benefits, such as increased employment earnings, are already expressed in dollar values and thus the conversion factor is 1. However, other benefits, like reduced days with medical problems, need to be converted to monetary values. The monetary conversion factors were obtained from the literature and are summarized in Table 2 [22].

**Table 2:** Benefit prediction functions

	Changes in	Prediction functions	Adj. R^2^	Baseline values	Monetary conversion factors
Inpatient	Alcohol consumption	32.143–0.975*(BASELINE ALCOHOL CONSUMPTION)	0.779	$150.75	1.00
	Employment earning	66.806–0.935*(BASELINE EMPLOYMENT EARNING) + 1354.584*MD	0.532	$145.40	1.00
	Days with medical problem	9.621–0.678*(BASELINE MEDICAL PROBLEM) + 71.633*PDTR	0.346	13.51 days	$26.44/day
	Days with psychological problem	10.648–0.599*(BASELINE PSYCHOLOGICAL PROBLEM)-9.659*RN	0.303	14.96 days	$10.21/day
	Days with drug problem	3.245–0.929*(BASELINE DRUG PROBLEM)-9.146*LVN	0.735	10.59 days	$18.33/day
	Days with alcohol problem	2.588–0.881*(BASELINE ALCOHOL PROBLEM)	0.578	12.35 days	$18.33/day
	Days in controlled environment	7.721–0.925*(BASELINE CONTROLLED ENVIRONMENT)-18.456*SOCWK	0.337	7.77 days	$18.33/day
Residential	Alcohol consumption	26.321–0.954*(BASELINE ALCOHOL CONSUMPTION)-0.093*(TREATMENT LENGTH) +144.574*PDTR	0.781	$93.41	1.00
	Employment earning	117.253–0.613*(BASELINE EMPLOYMENT EARNING) + 1025.813*PSYCH + 452.924*RN	0.222	$214.47	1.00
	Days with medical problem	7.787–0.615*(BASELINE MEDICAL PROBLEM) + 14.752*CLERC	0.298	11.47 days	$26.44/day
	Days with psychological problem	6.983–0.714*(BASELINE PSYCH PROBLEM) + 17.109*PDTR-82.503*RESMD	0.380	10.63 days	$10.21/day
	Days with drug problem	0.722–0.834*(BASELINE DRUG PROBLEM) + 15.673*PDTR	0.633	7.01 days	$18.33/day
	Days with alcohol problem	1.947–0.875*(BASELINE ALCOHOL PROBLEM)	0.600	8.87 days	$18.33/day
	Days in controlled environment	2.487–0.909*(BASELINE CONTROLLED ENVIRONMENT) + 0.081*(TREATMENT LENGTH) +9.428*LVN	0.447	10.15 days	$18.33/day
Intensive outpatient	Alcohol consumption	20.851–0.993*(BASELINE ALCOHOL CONSUMPTION)	0.773	$93.56	1.00
	Employment earning	117.034–0.679*(BASELINE EMPLOYMENT EARNING) + 910.813*PSYCH	0.312	$248.35	1.00
	Days with medical problem	10.014–0.573*(BASELINE MEDICAL PROBLEM)-11.941*RN	0.285	11.79 days	$26.44/day
	Days with psychological problem	7.564–0.718*(BASELINE PSYCH PROBLEM)-11.269*AIDES	0.380	11.84 days	$10.21/day
	Days with drug problem	0.954–0.833*(BASELINE DRUG PROBLEM)	0.625	6.87 days	$18.33/day
	Days with alcohol problem	2.635–0.869*(BASELINE ALCOHOL PROBLEM)-10.109*PSYCH	0.621	8.57 days	$18.33/day
	Days in controlled environment	3.759–0.88*(BASELINE CONTROLLED ENVIRONMENT) + 19.547*RN	0.362	8.21 days	$18.33/day
Standard outpatient	Alcohol consumption	21.502–0.937*(BASELINE ALCOHOL CONSUMPTION)-0.06*(TREATMENT LENGTH) +1.567*(TREATMENT INTENSITY) + 91.811*RESMD	0.833	$73.28	1.00
	Employment earning	249.071–0.837*(BASELINE EMPLOYMENT EARNING)-0.297*(TREATMENT LENGTH)	0.673	$315.62	1.00
	Days with medical problem	8.875–0.591*(BASELINE MEDICAL PROBLEM) + 0.327*(TREATMENT INTENSITY)-24.052*PDTR	0.304	12.55 days	$26.44/day
	Days with psychological problem	6.503–0.641*(BASELINE PSYCH PROBLEM) + 0.221*(TREATMENT INTENSITY) + 27.111*LVN	0.341	11.87 days	$10.21/day
	Days with drug problem	0.797–0.865*(BASELINE DRUG PROBLEM) + 2.572*PRACT + 17.56*LVN	0.625	4.88 days	$18.33/day
	Days with alcohol problem	2.812–0.809*(BASELINE ALCOHOL PROBLEM)-8.044*PSYCH-4.518*RN	0.531	6.97 days	$18.33/day
	Days in controlled environment	4.502–0.836*(BASELINE CONTROLLED ENVIRONMENT)-6.965*SOCWK + 14.917*OCTHR	0.405	6.49 days	$18.33/day

MD: % psychiatrists or mds; RESMD: % resident physicians or fellows; PRACT: % nurse practitioners or physicians assistants; PSYCH: % psychologists; socwk: % social workers; ADTHR: % addiction therapists; OCTHR: % recreational/occupational therapists; RN: % registered nurses; LVN: % licensed vocational/practical nurses; CLERC: % clerical staff; AIDES: % aides/technicians; PDTR: % paid trainees

The constraints for the optimization problem are stated in Eqs 2, 3, 4, 5, 6 and 7. Equation 2 indicates that the sum of all staff proportions should equal to 1. Equation 3 specifies that a program’s proportion for each staff type should be in the range of those observed in treatment programs, denoted by *x*_*observed*_. As two examples, standard outpatient program were run by a few as one staff member, an addiction therapist, and most standard outpatient programs did not have licensed vocational nurses as more than 10 % of total staff. Equation 4 specifies that the ratio of trainees (e.g., resident MDs or other paid trainees) to supervisors (e.g., MDs or psychologists) should fall within the observed ranges. In Eqs 5 and 6, *L* and *H* refer to treatment length in terms of days and treatment intensity in terms of hours per day, respectively, and we limited them to be within observed program ranges. We hypothesized that treatment length and intensity may change depending on staffing mix, because each type of staff provides different treatment services requiring different treatment lengths and intensities. Equation 7 restricts treatment benefits to within possible ranges. For example, if a patient suffers from drug problems for 20 days per month at admission, the treatment cannot possibly reduce the patient’s days of drug problems more than 20 days per month, nor can patients’ days of drug problems worsen to more than 30 days per month.

### Data

We derived prediction functions for benefits *B(x)* based on a VA Outcomes Monitoring Project (OMP) [23] database. The OMP was deemed an exempt project by the VA Palo Alto Health Care System/Stanford University IRB. The OMP sought to collect representative patient outcome data by randomly selecting a sample of programs and samples of their patients, and collected baseline and “6-month” follow-up data on patients in VA SUDTPs in three annual cohorts from FY01 to FY03 [23]. Compared with the previously mandated system-wide monitoring system, the OMP achieved a higher follow-up rate without paying patients for their participation (67 vs. 15–21 %) [23]. The actual follow-up point averaged 7.4 months (SD = 2.4 months) and the follow-up rate was 65.2 %. In all, 5548 patients in 55 standard outpatient, 36 intensive outpatient, 39 residential and 14 inpatient programs were assessed at baseline, and the patients in the methadone programs were excluded from the analysis. A brief self-report form [24] of the Addiction Severity Index (ASI) [25] was used to assess problems over the past 30 days in the seven ASI domains: alcohol use, drug use, psychiatric, medical, legal, family/social relationship, and employment problems. A cost-benefit analysis guideline for addiction treatment using ASI recommends including the following variables as benefits: reduced number of days in a controlled environment (medical, psychiatric, residential or hospital substance abuse treatment program), reduced number of days experiencing medical or psychiatric problems, increased income received from employment, reduced money spent on alcohol or drugs, and reduced number of days engaged in illegal activities [22].

Unfortunately, the self-report ASI form did not include the items to assess some key benefit variables, such as money spent on drugs and number of days engaged in illegal activities of the original ASI form. Thus, the analysis here may underestimate the benefits from treatment programs and may explain why the net benefits are estimated negative for some existing programs. In addition, the self-report form did not specify the type of controlled environment for each period in an environment; instead, it asked about how long a respondent stays in all types of controlled environments and whether the respondent stays in one or more controlled environment during the last 30 days. Thus, we could not use separate monetary conversion factors for each type of controlled environments, and we needed to calculate a ‘composite’ conversion factor for all types of controlled environments. We counted responses to each type of controlled environments and calculated changes in those responses between baseline and follow-up. We used the response changes for each type of controlled environment as weights to calculate a weighted arithmetic mean of the monetary conversion factors of all types of controlled environments, a ‘composite’ factor to convert reductions in days in all types of controlled environments into monetary benefits from a treatment.

The OMP also assessed VA SUDTPs’ characteristics, staff mix and services delivered via a program survey [23]. The survey gathered additional information on staff (e.g., time spent in group and individual treatment) to better assess program costs [23].

### Influence diagrams

Influence diagrams [26, 27] were used to specify likely potential relationships among variables affected by staffing mix and highlight each treatment program’s characteristics. In influence diagrams, a decision, such as staffing mix, is represented as a rectangle, and an uncertainty quantity, such as patient health status, is represented as an oval. A double oval represents a variable that is a deterministic function of its inputs, such as hourly staffing cost, and a diamond refers to the objective function, such as net benefit. Arcs represent possible conditional dependence among quantities [26, 27]. For example, having no arc from staffing mix to total staffing cost per patient indicates that they are conditionally independent, given hourly staffing costs.

**Fig. 1:**
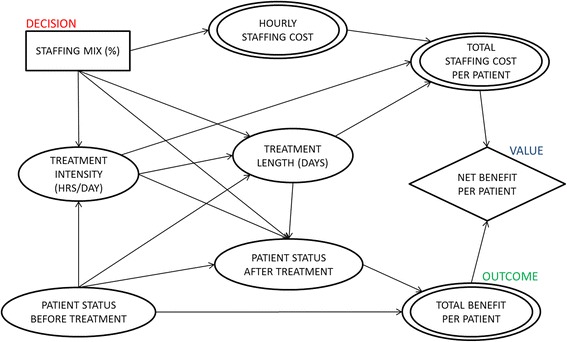
Influence diagram for VA SUD treatment program assuming no conditional independence

We formulated the hypothetical influence diagram in Fig. 1 based on the variables that are assessed in the OMP database assuming no conditional independence. The hypothetical model shows how staffing mix is believed to affect patient status after treatment. We allowed treatment length and intensity to change depending on the staffing mix. We also allowed treatment intensity and length to affect patient status after treatment and those treatment factors to be tailored to the baseline characteristics of patients (e.g., more severe patients would be treated longer). Treatment length was allowed to depend on treatment intensity (e.g., more intense treatments might be provided for shorter periods). Total staffing costs were calculated by multiplying hourly staffing cost, treatment intensity, and treatment length. Benefits from treating a patient were calculated by subtracting baseline patient status from patient status at follow-up, and total benefits were calculated by multiplying benefits with the average follow-up point (e.g., 7.4 months) [22]. Net benefits are total benefits less total staffing costs.

Based on the hypothetical influence diagram, we examined the correlations among variables for each type of program, and whenever there was any nonzero correlation between two variables at the significance level of 0.05, we included an arc between them. The correlations among variables also can be found from prediction functions in the following section; however, influence diagrams graphically present the relations among variables more clearly.

### Prediction functions

**Fig. 2:**
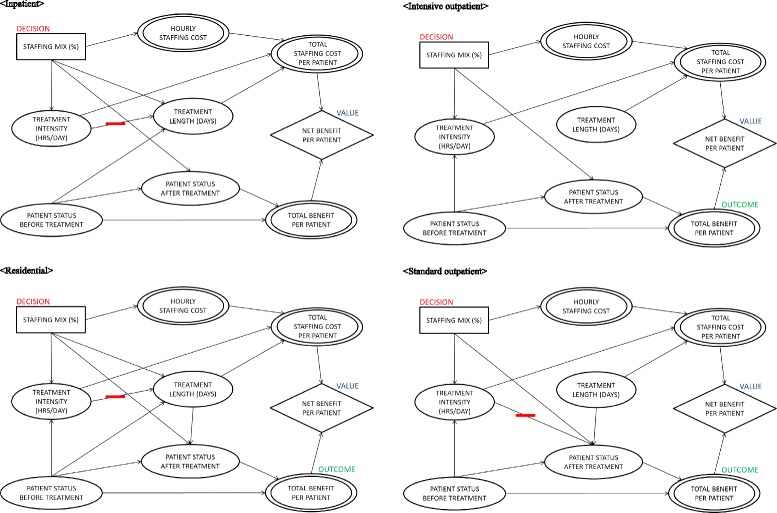
Influence diagrams of VA SUD treatment programs

We derived prediction functions for benefits (Table 2) by using a stepwise method. The same covariates were included in prediction functions derived from other methods (e.g., backward method), and coefficients of the covariates were almost identical. For some types of treatment programs, baseline patient status and staff variables were the only covariates to predict benefits, but for others treatment intensity and length have predictive power as presented in Fig. 2. We did not find any significant correlations among the staff variables and did not include any interactions between staff variables in deriving prediction functions. For all benefit prediction functions, baseline patient status appeared to be a major benefit driver, and if a patient came to treatment when he or she was in a more serious status, changes in patient status between before and after treatments were likely to be larger (as would be expected from regression to the mean). For the base analysis, we used average patient status values and then varied them in sensitivity analyses. We also derived prediction functions for staffing cost factors (treatment intensity and treatment length) (Table 3) by using a stepwise method and verified them with other methods. Hourly staffing cost is a weighted arithmetic mean of different types of staffs’ actual costs [28], and only treatment intensity and treatment length needed to be predicted with staff and baseline patient status covariates (Table 3). Treatment length for standard and intensive outpatient programs could not be predicted with enough predictive power (e.g., adjusted R^2^ less than 0.10); thus, the average constant values of those cost factors were used for the analysis. All statistical analyses were conducted using PASW Statistics 18, Release Version 18.0.0 (SPSS, Inc., 2009, Chicago, IL, www.spss.com).

**Table 3:** Prediction functions for treatment intensity and treatment length

		Cost factor	Prediction function	Adj. R^2^
24 h care	Inpatient	Treatment intensity (hours/day)	12.146–452.776*RESMD-8.568*RN + 124.438*CLERC + 187.722*PDTR-69.657*SOCWK-22.342*PRACT + 4.941*ADTHR-15.617*PSYCH + 268.324*OCTHR-95.138*MD-31.492*AIDES	1.000
		Treatment length (days)	−28.168 + 118.794*PSYCH + 210.522*SOCWK + 86.483*RN-632.262*PDTR + 176.915*PRACT + 0.281*(BASELINE CONTROLLED ENVIRONMENT)	0.469
	Residential	Treatment intensity (hours/day)	0.594 + 161.368*RESMD + 7.160*AIDES + 7.131*RN + 10.740*PRACT + 11.241*MD-4.762*SOCWK + 8.193*OCTHR + 4.170*PSYCH −0.014*(BASELINE DRUG PROBLEM)-0.012*(BASELINE CONTROLLED ENVIRONMENT)	0.517
		Treatment length (days)	65.688–208.151*PRACT + 392.280*OCTHR + 257.058*MD-892.674*RESMD-191.554*PDTR-72.917*CLERC-57.393*RN −57.240*SOCWK + 0.582*(BASELINE CONTROLLED ENVIRONMENT)-0.008*(BASELINE EMPLOYMENT EARNING) −0.248*(BASELINE MEDICAL PROBLEM)	0.195
Outpatient	Intensive	Treatment intensity (hours/day)	5.002 + 23.378*PRACT-10.074*LVN-20.253*PSYCH + 7.961*PDTR-7.992*SOCWK + 7.700*OCTHR + 3.800*MD-6.926*RESMD −5.657*AIDES-3.417*ADTHR + 0.008*(BASELINE PSYCH PROBLEM)	0.957
		Treatment length (days)	97.435–0.797*(BASELINE DRUG PROBLEM)-103.899*RN-110.129*RESMD	0.026
	Standard	Treatment intensity (hours/day)	3.604 + 8.185*PRACT-3.924*ADTHR-4.250*AIDES-4.325*CLERC + 6.289*RESMD + 4.266*PSYCH + 4.276*OCTHR +0.209*(10^-3)*(BASELINE EMPLOYMENT EARNING)	0.372
		Treatment length (days)	59.413 + 0.819*(BASELINE CONTROLLED ENVIRONMENT) + 333.825*PDTR + 45.783*PRACT + 310.603*LVN	0.022

MD: % psychiatrists or mds; RESMD: % resident physicians or fellows; PRACT: % nurse practitioners or physicians assistants; PSYCH: % psychologists; SOCWK: % social workers; ADTHR: % addiction therapists; OCTHR: % recreational/occupational therapists; RN: % registered nurses; LVN: % licensed vocational/practical nurses; CLERC: % clerical staff; AIDES: % aides/technicians; PDTR: % paid trainees

## Results

### VA program characteristics

Based on data from OMP [23], Table 4 provides the characteristics of patients admitted to each type of treatment program, as well as information on treatment intensity (i.e., hours per day) and length (i.e., days) of those programs. The programs are listed in decreasing order of the severity of patients’ conditions, treatment intensity, and (more or less) in increasing order of the treatment length. Not surprisingly, standard and intensive outpatient programs provided more lengthy treatment services.

**Table 4:** Patient status when admitted to four type of treatment programs, and treatment intensity and length of the programs

		Inpatient	Residential	Intensive outpatient	Standard outpatient
		(*N* = 603)	(*N* = 1763)	(*N* = 1374)	(*N* = 1808)
		Mean (SD)	Mean (SD)	Mean (SD)	Mean (SD)
Patient status when admitted	Employment earnings ($/month)	145.40 (426.99)	214.47 (618.91)	248.35 (634.06)	315.62 (914.15)
	Alcohol consumption ($/month)	150.75 (264.02)	93.41 (202.06)	93.56 (216.71)	73.28 (220.18)
	Days with medical problems (days/month)	13.51 (13.25)	11.47 (13.08)	11.79 (13.15)	12.55 (13.32)
	Days with psychological problems (days/month)	14.96 (12.14)	10.63 (11.92)	11.84 (12.22)	11.87 (12.27)
	Days with drug problems (days/month)	10.59 (12.45)	7.01 (10.74)	6.87 (10.66)	4.88 (9.60)
	Days with alcohol problems (days/month)	12.35 (12.22)	8.87 (11.31)	8.57 (11.36)	6.97 (10.55)
	Days in controlled environments (e.g. jail) (days/month)	7.77 (9.11)	10.15 (11.11)	8.21 (10.22)	6.49 (10.32)
Treatment administration	Treatment intensity (hours/day)	6.10 (3.56)	3.88 (3.09)	3.26 (4.77)	3.03 (3.63)
	Treatment length (days)	33.27 (40.03)	57.98 (62.48)	74.24 (93.33)	72.56 (113.03)

### Influence diagrams

Figure 2 summarizes the influence diagrams for the four types of SUDTPs. In all program types, staffing mix was associated with treatment intensity and patient outcome after treatment, but staffing mix was related to treatment length only in inpatient and residential programs. This implies that treatment intensities and patient outcomes of all SUDTPs may depend on their staffing mixes which also determine treatment lengths of inpatient and residential programs. Patient status before treatment was associated with treatment intensity in residential, intensive outpatient, and standard outpatient programs, suggesting that these types of programs tailor treatment intensity based on patient status at the start of care. Conversely, there was no relationship between patient status before treatment and treatment length in intensive outpatient and standard outpatient programs. Presumably, these programs titrate treatment exposure based on patient response to care (e.g., success in achieving initial abstinence or medical stability), rather than patients’ initial state. In other words, these outpatient programs try to keep patients in treatment until they get better, rather than setting treatment duration based on illness severity at intake. Notably, treatment intensity and length were not associated with patient status at follow-up within intensive outpatient and inpatient programs. Thus, for these program types, lengthier treatments were not associated with greater improvement in patients’ outcomes, but they were more costly. For inpatient and residential programs, treatment length was negatively associated with treatment intensity (e.g., more intense treatments were provided for shorter periods), whereas there was no significant correlation for intensive and standard outpatient programs.

### Actual versus suggested staffing mix for treatment programs

**Fig. 3:**
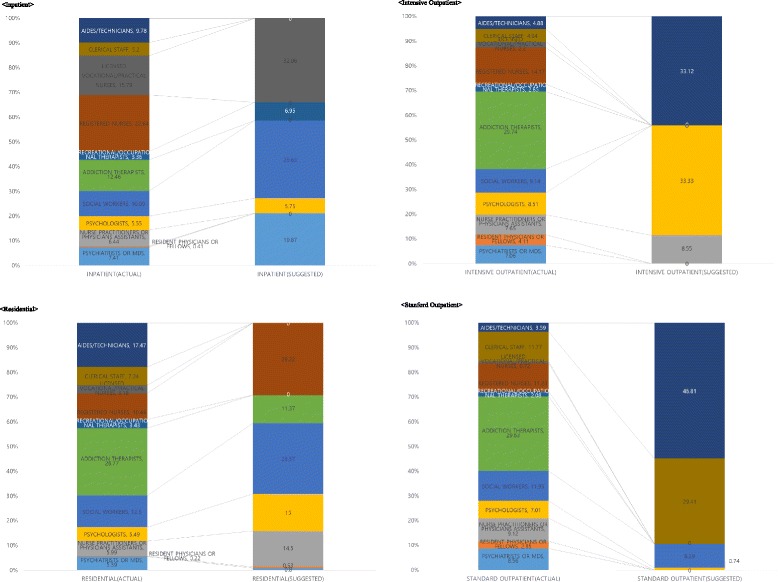
Actual and suggested optimal staffing mix for VA SUDTPs

The actual staffing mixes in FY01-FY03 for different types of SUDTPs were not significantly different, although there was substantial variation in staffing mix within each type of SUDTP (Table 5). Table 5 summarizes actual staffing patterns in FY01-FY03 and compares them to staffing mixes suggested by solving the optimization problem for the four types of SUDTPs. The summaries and comparisons in Table 5 are visualized in Fig. 3.

**Table 5:** Actual and suggested optimal staffing mix for VA SUDTPs

Staff	Inpatient		Residential		Intensive outpatient		Standard outpatient	
	Actual	Suggested	Actual	Suggested	Actual	Suggested	Actual	Suggested
	FY01-FY03		FY01-FY03		FY01-FY03		FY01-FY03	
	mean % (SD)	%	mean % (SD)	%	mean % (SD)	%	mean % (SD)	%
MD	7.41 (5.11)	19.87	5.39 (3.97)	0.80	7.06 (8.29)	0.00	8.56 (9.53)	0.00
RESMD	0.41 (1.24)	0.00	0.22 (0.85)	0.53	4.11 (7.93)	0.00	2.85 (5.60)	0.00
PRACT	6.44 (9.29)	0.00	5.99 (6.60)	14.50	7.65 (15.58)	8.55	9.12 (20.44)	0.00
PSYCH	5.55 (9.65)	5.75	5.49 (4.46)	15.00	8.51 (8.74)	33.33	7.01 (7.53)	0.74
SOCWK	10.09 (8.60)	29.63	12.50 (7.82)	28.57	9.14 (8.32)	0.00	11.95 (12.30)	8.29
ADTHR	12.46 (11.16)	0.00	26.77 (23.25)	11.37	29.74 (17.56)	0.00	29.63 (25.86)	0.00
OCTHR	3.36 (2.76)	6.95	3.43 (3.22)	0.00	2.85 (4.19)	0.00	2.08 (5.11)	0.00
RN	22.64 (14.91)	0.00	10.44 (9.18)	29.22	14.17 (10.22)	0.00	11.23 (12.34)	0.00
LVN	15.79 (12.95)	32.06	3.18 (8.23)	0.00	2.20 (6.66)	0.00	0.72 (2.49)	0.00
CLERC	5.20 (3.32)	0.00	7.24 (5.61)	0.00	4.94 (4.56)	0.00	11.77 (9.61)	29.41
AIDES	9.78 (12.96)	0.00	17.47 (20.16)	0.00	4.88 (9.73)	33.12	3.59 (12.90)	46.81
PDTR	0.88 (1.91)	5.75	1.88 (4.36)	0.00	4.75 (8.07)	25.00	1.49 (3.13)	14.75
Benefits ($)	5989.96	14,808.92	5888.00	17,904.42	3179.05	10,319.39	1330.67	2136.45
Costs ($)	6286.93	298.46	7408.10	161.05	9072.54	1012.52	8140.95	664.27
Net benefits ($)	−296.97	14,510.46	−1520.10	17,743.37	−5893.49	9306.87	−6810.28	1472.18

MD: % psychiatrists or mds; RESMD: % resident physicians or fellows; PRACT: % nurse practitioners or physicians assistants; PSYCH: % psychologists; SOCWK: % social workers; ADTHR: % addiction therapists; OCTHR: % recreational/occupational therapists; RN: % registered nurses; LVN: % licensed vocational/practical nurses; CLERC: % clerical staff; AIDES: % aides/technicians; PDTR: % paid trainees

The suggested staffing mix of inpatient programs includes more MDs or psychiatrists than other treatment programs, likely reflecting the medical orientation of such programs. However, MDs or psychiatrists typically are more expensive to employ than other types of prescribers (e.g., resident MDs and nurse practitioners). In contrast, for residential and intensive outpatient programs, suggested staffing mixes are redistributed to less expensive staff members who can perform similar functions. The model suggests it would be cost-effective to increase proportions of nurse practitioners who might function similarly to MDs or psychiatrists as prescribers. Interestingly, such changes increase patient benefits in addition to reducing costs. For residential and intensive outpatient programs, more psychologists were recommended. Although psychologists are more expensive than other psychosocial rehabilitators (e.g., social workers, addiction therapists, and occupational therapists), they more strongly increase patients’ earnings from employment and reduce their days with alcohol problems, with the value of those benefits being larger than the costs to achieve them. The suggested staffing mix of inpatient programs would involve hiring more licensed vocational nurses than registered nurses; however, the recommendation for residential programs is the opposite.

In intensive outpatient programs, although psychologists are more expensive than other psychosocial rehabilitators, they increase patients’ earnings from employment and reduce their days with alcohol problems, with the value of those benefits outweighing the costs to achieve them. The suggested staffing of standard outpatient programs would involve hiring more administrative staff and paid trainees, potentially indicating that during the FY01-03 period some more expensive VA clinical staff may have been performing functions that could have been effectively completed by less costly administrative staff or trainees.

At the suggested staffing mixes, net benefits from the treatment programs become positive. While benefits from treating a patient would be expected to increase, total staffing costs would decrease drastically under the suggested staffing mixes. Suggested staffing mixes are directed at minimizing total treatment hours in addition to hourly staffing costs, while maintaining treatment effectiveness.

### Sensitivity analyses

Sensitivity analyses were conducted to check the robustness of the results described in the previous section. First, we varied the monetary conversion factors for days with drug and alcohol problems. The self-report ASI asked only about number of days having drug or alcohol problems, but it did not specify whether those problems are physical or psychological ones. Thus, we used an average value of the monetary conversion factors for medical and psychological problems from prior research [21] for the base analysis, and then varied it within both ranges in the sensitivity analyses. The sensitivity analysis showed that the optimal staffing mixes for all treatment programs are insensitive to changes in the monetary conversion factor. In addition, we varied baseline severity of addiction problems in treatment patients; in addition to the mean values of addiction problem severity, we used lower (2.5 %) and higher (97.5 %) patient baseline addiction problem severity values derived from bootstrapping. The sensitivity analysis demonstrated that the originally calculated optimal staffing mixes are not sensitive to changes in baseline patient addiction problem severity, although, as expected, we observed that when a patient starts with more serious addiction problems, the derived benefits (and net benefits) from treating the patient are greater.

## Discussion

Recommended staffing mixes for substance use disorder treatment programs differ depending on the type of program. Given the differences in focus and the intended population of the programs, this was expected. The staffing requirements or licensure/accreditation requirement for each type of programs were already reflected since observed ranges were used as constraints in the optimization problems. With the modeled optimized staffing mixes, we found that treatment intensity and length would be substantially shorter than average values. Thus, optimizing staffing mixes could not only increase net benefits of the treatment programs, but could also reduce treatment time. Reductions in treatment time would allow VA SUDTPs to treat more patients with the same number of staff, bringing SUD treatment benefits to more Veteran patients and potentially improving access and reducing wait-times for care.

Encouragingly, recommended outpatient staff mix recommendations appear in line with treatment goals at various stages of treatment. Specifically, a main goal of intensive outpatient care is to help SUD patients who are actively using or at high risk of relapse to achieve an initial period of stable abstinence. Evidence-based SUD psychotherapy and pharmacotherapy protocols would thus be expected to dominate effective care directed toward this goal [29, 30]. We recommend staffing these programs with psychologists, aides and nurse practitioners/physician assistants (NPs/PAs), and eliminating other psychosocial rehabilitators, medical doctors (MDs) and other nursing staff. Notably, psychologists should be optimally trained to deliver the evidence-based psychotherapy protocols shown in randomized controlled trials to be effective for achieving abstinence. Unlike other nurses, NPs/PAs are able to prescribe medications for alcohol use disorder pharmacotherapy and co-morbid mental health disorders, and can do so at lower cost than MDs. Because of their lower cost, NPs/PAs may be able to spend more time with patients than MDs, which may be beneficial for developing treatment plans and delivering treatments to modify behaviors.

In contrast, a main goal of standard outpatient programs is to help patients maintain abstinence once they are stabilized. Maintaining case-management and contact with recovery-focused services, such as mutual help groups, and addressing psychiatric co-morbidities and psychosocial issues that may increase risk of relapse has been shown to be important for maintaining abstinence [31–36]. In these programs, our model suggests staffing primarily with social workers with lower percentages of psychologists and higher percentages of administrative staff. Monitoring abstinence, encouraging recovery-focused activities, and case management are all within the training of social workers and these staff are substantially less expensive than psychologists.

Compared to other methods (e.g., measuring clinician productivity), our approach to optimal staffing captures the importance of staff members, such as clerks, who do not provide direct patient care for achieving both greater efficiency and better patient outcomes. Results of a recent survey of VA SUDTPs suggest that support staff may be crucially important for retaining other program staff and thus maintaining efficient delivery of SUD treatment. Specifically, the FY10 VA Drug and Alcohol Program Survey found that after retirement and promotion, the most frequent reason cited for loss of staff from SUD treatment programs in the previous year was “excess administrative burden” [37]. Increasing support staff may be an efficient and effective intervention to help clinical staff focus more on treating SUD patients, potentially improving treatment outcomes while limiting program costs.

The recommended intensive outpatient program staffing eliminates medical doctors, including psychiatrists and residents, from these programs and greatly increases staffing with paid trainees and aides. Although medical doctors had some beneficial impact on psychological outcomes, they did not have a significant impact on alcohol and substance use and other outcomes considered in these analyses. We note that these findings must be interpreted within the context of practice patterns in VA SUDTPs in FY01-FY03. Buprenorphine-based opioid agonist treatment is a highly effective outpatient treatment for opioid dependence that was approved by the U.S. Federal Drug Administration in 2002, and NP/PAs cannot prescribe buprenorphine for the treatment of opioid dependence (e.g. SAMHSA, DATA 2000 http://buprenorphine.samhsa.gov/data.html). At the time the data analyzed here were collected, SUDTPs rarely provided effective pharmacotherapies for substance use disorders to patients. Opioid agonist treatment for opioid dependence was offered in separate methadone maintenance clinics and pharmacotherapy for alcohol use disorders was provided to only 3 % of patients with an alcohol use disorder nationally [38]. Increasing use of evidence-based pharmacotherapies by medical doctors may increase the beneficial effects of such staff on substance use and associated problems (e.g., illegal activity and risk of being in a controlled environment). Reevaluation of the recommendation to eliminate medical doctors in intensive outpatient programs is warranted in programs where opioid agonist treatment with buprenorphine and effective alcohol pharmacotherapies are regularly prescribed. Nevertheless, these findings emphasize that, at least in the absence of use of SUD pharmacotherapies, medical doctors do not provide benefits that justify their cost in programs focused on early outpatient stabilization and achieving initial abstinence. Likewise, since FY01-FY03, VA has greatly increased focus on training and provision of evidence-based psychotherapies within its mental health programs. Use of these evidence-based therapies may substantially increase treatment benefits of patients, and paid trainees and aides may not be able to reliably deliver these therapies. Additionally, not all VA medical centers have training programs, and thus, although the model recommendations are within the range observed in VA SUD programs in FY01-FY03, they may need to be reconsidered in the context of current care practices and facility context.

Reconfiguring staffing mix in SUDTPs may be feasible through natural attrition. In general, turnover among mental health service providers is high, typically between 25–50 % per year, which, although detrimental for treatment access, implementation of evidence-based treatments, and quality of care, provides opportunities to restructure treatment program staffing relatively rapidly [39–46]. In addition, many VA employees are entering retirement age. Beneficially, many medical centers include clinical training programs that together train over 100,000 health professionals per year. For example, 70 % of U.S. physicians have received at least some of their training at VA hospitals [47]. These training programs provide a large pool of new health professionals who could be rapidly integrated into VA SUDTPs. Therefore, it may be feasible to implement optimized staffing mixes in real-world settings.

## Limitations

First, this analysis is based upon program staffing and practice patterns and patient outcomes in FY01-FY03. Although this time period is that for which the most recent VA system-wide patient substance use disorder treatment outcomes data are available, the system has been changed since that time by numerous national, regional, and local quality improvement efforts, dramatic expansion of mental health staffing, increases in VA enrollees, and gradual shifts in the demographics and mix of substance use disorder diagnoses among VA patient populations [48]; in addition, there has been many new innovations that had been implemented such as the use of computer simulations to help people who are diagnosed with PTSD since the period. Ideally, the analyses reported here would be repeated using outcomes data from more recent SUDTP patients. Second, the present analyses include only VA SUDTPs and the findings may not generalize to other treatment settings. VA SUDTPs generally treat a population predominated by older male patients with chronic substance use disorders and high rates of psychiatric co-morbidity. Third, since FY01-FY03, there has been a trend toward restructuring SUDTPs at VA medical centers, such that inpatient, residential, intensive outpatient and standard outpatient care is combined into a single umbrella program. It is not immediately clear from these analyses how to optimize staff mix in such mixed-purpose programs; however, one might reasonably expect the staffing recommendations to hold for components of these mixed programs with the same treatment focus and goals as the program types studied here. Finally, these analyses lacked some estimates such as reductions in spending on drugs and in time engaged in illegal activities in the benefit calculations. Thus, these analyses almost certainly underestimate treatment benefits at 6 months. These analyses also do not include estimates of longer-term benefits since the actual follow-up of patients is very short (e.g., 7.4 months), which may be particularly important for optimizing staffing mix in standard outpatient programs, where the focus is on relapse prevention and fostering recovery.

## Conclusions

There has been no systematic empirical method for staffing SUDTPs. The approach reported here provides a rational basis for staffing SUDTPs in a way that efficiently maximizes benefits for patients. This approach also provides a framework to systematically incorporate characteristics of the intended patient population into staffing decisions at SUDTPs. Given the availability of collected patient outcomes data, this approach provides a systematic method to guide SUDTP staffing based on the patient benefits, staffing costs, and dynamics of the treated patient population.

## Abbreviations

ASI: addiction severity index
FY: fiscal year
GRG: generalized reduced gradient
MDs: medical doctors
NPs/PAs: nurse practitioners/physician assistants
OMP: outcomes monitoring project
SUD: substance use disorder
SUDTPs: substance use disorder treatment programs
VA: Department of Veterans Affairs

## References

[1] Wagner TH, Sinnott P, Siroka AM (2011). Mental health and substance use disorder spending in the department of veterans affairs, fiscal years 2000–2007. Psychiatr Serv.

[2] Dalton A, McKellar J (2008). Health services for VA substance use disorder patients: comparison of utilization fiscal years 2008, 2007, 2006, 2005, and 2002.

[3] Tracy S, Tavakoli S, Stolpner S, Trafton J (2010). The department of veterans affairs substance use disorder treatment system: results of the 2008 drug and alcohol program survey.

[4] Roose RJ, Kunins HV, Sohler NL, Elam RT, Cunningham CO (2008). Nurse practitioner and physician assistant interest in prescribing buprenorphine. J Subst Abuse Treat.

[5] Stein DM, Lambert MJ (1995). Graduate training in psychotherapy: are therapy outcomes enhanced?. J Consult Clin Psychol.

[6] Lasdon LS, Fox R, Ratner M (1973). Nonlinear optimization using the generalized reduced gradient method. Tech. Memo. 325.

[7] Lasdon LS, Waren AD, Jain A, Ratner M (1978). Design and testing of a generalized reduced gradient code for nonlinear programming. ACM Trans Math Softw.

[8] Fylstra D, Lasdon L, Watson J, Waren A (1998). Design and use of the microsoft excel solver. Interfaces.

[9] Kent DJ, Shachter RD, Sox HC, Ng HS, Shortliffe LD, Moynihan S (1989). Efficient scheduling of cystoscopies in monitoring for recurrent bladder cancer. Med Decis Making.

[10] Kent DL, Nease RA, Sox HC, Shortliffe LD, Shachter RD (1991). Evaluation of nonlinear optimization for scheduling of follow-up cystocopies to detect recurrent bladder cancer. Med Decis Making.

[11] Brandeau ML, Zaric GS (2009). Optimal investment in HIV prevention programs: more is not always better. Health Care Manag Sci.

[12] Zaric GS, Brandeau ML (2001). Optimal investment in a portfolio of HIV prevention programs. Med Decis Making.

[13] Richter A, Brandeau ML (1999). An analysis of optimal resource allocation for HIV prevention among injection drug users and nonusers. Med Decis Making.

[14] Armbruster B, Brandeau ML (2007). Optimal mix of screening and contact tracing for endemic diseases. Math Biosci.

[15] Barreto A, Barros MO, Werner CML (2008). Staffing a software project: a constraint satisfaction and optimization-based approach. Comput Oper Res.

[16] Kapur P, Ngo-The A, Ruhe G, Smith A (2008). Optimized staffing for product releases and its application at Chartwell Technology. J Softw Maint Evol-R.

[17] Gagnon RJ, Krasner JD (1990). The optimal mix of internal/external engineering staff and equipment with different levels of capability and performance. IEEE T Eng Manage.

[18] Whitt W (2006). Staffing a call center with uncertain arrival rate and absenteeism. Prod Oper Manag.

[19] Andrews B, Parsons H (1993). Establishing telephone-agent staffing levels through economic optimization. Interfaces.

[20] Warner DM, Prawda J (1972). A mathematical programming model for scheduling nursing personnel in a hospital. Manage Sci.

[21] Franz LS, Baker HM, Leong GK, Rakes TR (1989). A mathematical model for scheduling and staffing multiclinic health regions?. Eur J Oper Res.

[22] French MT, Salome’ HJ, Sindelar JL, McLellan AT (2002). Benefit-cost analysis of addiction treatment: methodological guidelines and empirical application using the DATCAP and ASI. Health Serv Res.

[23] Tiet QQ, Byrnes HF, Barnett P, Finney JW (2006). A practical system for monitoring the outcomes of substance use disorder patients. J Subst Abuse Treat.

[24] Rosen CS, Henson BR, Finney JW, Moos RH (2000). Consistency of self-administered and interview-based addiction severity index composite scores. Addiction.

[25] McLellan AT, Kushner H, Metzger D, Peters R, Smith I, Grissom G (1992). The fifth addition of the addiction severity index. J Subst Abuse Treat.

[26] Shachter RD (1986). Evaluating influence diagrams. Oper Res.

[27] Howard RA (1971). Proximal decision analysis. Manage Sci.

[28] Smith MW, King SS (2010). A guide to estimating wages of VHA employees – wage tables FY2000 to FY2008. Technical report 25 supplement.

[29] McGovern MP, Carroll MK (2003). Evidence–based practices for substance use disorders. Psychiatr Clin N Am.

[30] Department of Veterans Affairs and Department of Defense. VA/DoD clinical practice guideline for management of substance use disorders. 2009. http://www.healthquality.va.gov/guidelines/MH/sud/. Accessed 22 Nov 2015.

[31] Ouimette P, Moos RH, Finney JW (2003). PTSD treatment and 5-year remission among patients with substance use and posttraumatic stress disorders. J Consult Clin Psych.

[32] Drake RE, Wallach MA, McGovern MP (2005). Future directions in preventing relapse to substance abuse among clients with severe mental illnesses. Psychiatr Serv.

[33] McKay JR (2006). Continuing care in the treatment of addictive disorders. Curr Psychiatry Rep.

[34] Kelly JF, Stout R, Zywiak W, Schneider R (2006). A 3-year study of addiction mutual-help group participation following intensive outpatient treatment. Alcohol Clin Exp Res.

[35] Moos RH, Moos BS, Timko C (2006). Gender, treatment and self-help in remission from alcohol use disorders. Clin Med Res.

[36] Ouimette P, Coolhart D, Funderburk JS, Wade M, Brown PJ (2007). Precipitants of first substance use in recently abstinent substance use disorder patients with PTSD. Addict Behav.

[37] Tracy S, Stolpner S, Rogers J, Tavakoli S, Trafton J (2011). The department of veterans affairs substance use disorder treatment system: results of the 2010 drug and alcohol program survey.

[38] Harris AH, Kivlahan DR, Bowe T, Humphreys KN (2010). Pharmacotherapy of alcohol use disorders in the veterans health administration. Psychiatr Serv.

[39] Woltmann EM, Whitley R, McHugo GJ, Brunette M, Torrey WC, Coots L (2008). The role of staff turnover in the implementation of evidence-based practices in mental health care. Psychiatr Serv.

[40] Ben-Dror R (1994). Employee turnover in community mental health organization: a developmental stages study. Community Ment Health J.

[41] Kamis-Gould E, Staines G (1986). Manpower information and the community mental health system. Hosp Community Psychiatry.

[42] Jayaratne S, Chess WA (1984). Job satisfaction, burnout, and turnover: a national study. Soc Work.

[43] Siefert K, Jayaratne S, Chess WA (1991). Job satisfaction, burnout, and turnover in health care social workers. Health Social Work.

[44] Aarons GA, Sawitzky AC (2006). Organizational climate partially mediates the effect of culture on work attitudes and staff turnover in mental health services. Adm Policy Ment Health.

[45] Gallon SL, Gabriel RM, Knudsen JRW (2003). The toughest job you’ll ever love: a pacific northwest treatment workforce survey. J Subst Abuse Treat.

[46] Glisson C, Durick M (1988). Predictors of job satisfaction and organizational commitment in human service organizations. Admin Sci Quart.

[47] Remarks by Deputy Secretary Sloan Gibson, presented to the West Point Society of DC on September 23, 2015. http://www.va.gov/opa/speeches/2015/09_23_2015.asp, Accessed October 31, 2015.

[48] Tracy S, Tavakoli S, Stolpner S, Trafton JA (in press) Treating substance use disorders within the veterans affairs health care system. In Veterans Health Reference, Miller, T (ed). Praeger: New York.

